# Therapeutic Efficacy of Orally Administered Nitrofurantoin against Animal African Trypanosomosis Caused by *Trypanosoma congolense* Infection

**DOI:** 10.3390/pathogens11030331

**Published:** 2022-03-09

**Authors:** Keisuke Suganuma, David D. N’Da, Ken-ichi Watanabe, Yusuke Tanaka, Ehab Mossaad, Afraa Elata, Noboru Inoue, Shin-ichiro Kawazu

**Affiliations:** 1 OIE Reference Laboratory for Surra, National Research Centre for Protozoan Diseases, Obihiro University of Agriculture and Veterinary Medicine, Obihiro 80-8555, Japan; ehabmssd7@gmail.com (E.M.); afraa31@hotmail.com (A.E.); ircpmi@obihiro.ac.jp (N.I.); 2 Research Center for Global Agromedicine, Obihiro University of Agriculture and Veterinary Medicine, Obihiro 080-8555, Japan; knabe@obihiro.ac.jp; 3 Centre of Excellence for Pharmaceutical Sciences (PHARMACEN), North-West University, Potchefstroom 2520, South Africa; david.nda@nwu.ac.za; 4 Veterinary Pathology, Obihiro University of Agriculture and Veterinary Medicine, Obihiro 080-8555, Japan; dondonobihiro@outlook.jp; 5 Department of Pathology, Parasitology and Microbiology, College of Veterinary Medicine, Sudan University of Science and Technology, Khartoum P.O. Box 204, Sudan; 6 Department of Preventive Medicine and Public Health, Faculty of Veterinary Medicine, University of Khartoum, Khartoum 13314, Sudan; 7 National Research Centre for Protozoan Diseases, Obihiro University of Agriculture and Veterinary Medicine, Obihiro 080-8555, Japan; skawazu@obihiro.ac.jp

**Keywords:** animal African trypanosomosis, nitrofurantoin, oral administration, trypanocidal drug, *Trypanosoma congolense*

## Abstract

Animal African trypanosomosis (AAT) leads to emaciation and low productivity in infected animals. Only six drugs are commercially available against AAT; they have severe side effects and face parasite resistance. Thus, the development of novel trypanocidal drugs is urgently needed. Nitrofurantoin, an antimicrobial, is used for treating bacterial urinary tract infections. Recently, we reported the trypanocidal effects of nitrofurantoin and its analogs in vitro. Furthermore, a nitrofurantoin analog, nifurtimox, is currently used to treat Chagas disease and chronic human African trypanosomiasis. Thus, this study was aimed at evaluating the in vivo efficacy of nitrofurantoin in treating AAT caused by *Trypanosoma congolense*. Nitrofurantoin was orally administered for 7 consecutive days from 4 days post-infection in *T. congolense*-infected mice, and the animals were observed for 28 days. Compared to the control group, the treatment group showed significantly suppressed parasitemia at 6 days post-infection. Furthermore, survival was significantly prolonged in the group treated with at least 10 mg/kg nitrofurantoin. Moreover, 100% survival and cure was achieved with a dose of nitrofurantoin higher than 30 mg/kg. Thus, oral nitrofurantoin administration has potential trypanocidal efficacy against *T. congolense*-induced AAT. This preliminary data will serve as a benchmark when comparing future nitrofurantoin-related compounds, which can overcome the significant shortcomings of nitrofurantoin that preclude its viable use in livestock.

## 1. Introduction

Animal African trypanosomosis (AAT) or Nagana, which is caused by *Trypanosoma congolense, Trypanosoma vivax*, or *Trypanosoma brucei brucei*, is prevalent in 36 sub-Saharan countries, consistent with tsetse (*Glossina* spp.) habitat [1,2]. On the other hand, other animal trypanosomoses, namely surra and dourine caused by *Trypanosoma evansi* and *Trypanosoma equiperdum*, respectively, are distributed worldwide because the transmission of these diseases is not directly dependent on specific biological vectors such as animal African trypanosomes [3,4,5]. The host range and severity of the disease are influenced by various factors, such as host animals and trypanosome strains. Hence, animal trypanosomosis caused by *T. congolense* is the most important animal disease, infecting a wide range of animals, including cattle, sheep, goats, and wildlife [6]. The endemicity of the disease causes severe economic loss in the livestock industry, especially in developing countries. Therefore, disease control is the most important issue in terms of improving animal productivity. Since trypanosomes frequently and randomly change their cell surface antigen, variant surface glycoprotein (VSG), it generally makes the development of an effective vaccine against trypanosomiasis difficult [7]. On the other hand, the vaccination efficacy of an invariant surface protein in *T. vivax* has been shown in a previous report [8]. Hence, this strategy, i.e., vaccination by an invariant protein, might be applicable to other trypanosome species.

Moreover, it is difficult to reduce vector density and trypanosome prevalence by using only vector control measures, such as insect traps, insecticide spraying, and sterile insect techniques in wide endemic areas [9]. To overcome the difficulty of the traditional tsetse control strategy, in the current tsetse control projects, new effective tsetse capture traps were developed [10,11,12]. Furthermore, an integrated approach, involving the combination of a baseline survey, aerial and ground spraying of insecticides, insecticide-impregnated targets, and the mass trypanocide treatment of domestic animals, could achieve a tsetse/trypanosomosis-free area in Burkina Faso [13]. In addition to vector control and mass drug administration, the main strategy for disease control includes both early diagnosis and the appropriate treatment of animal trypanosomosis in infected animals [14].

To date, only six drugs against animal trypanosomosis, namely diminazene, homidium bromide/chloride, isometamidium, quinapyramine sulfate/sulfate:chloride, suramin, and melarsomine, are available in the market and applied for the treatment and/or prophylactic use [1]. These drugs were discovered and have been used for the treatment of animal trypanosomosis for 30 to 100 years. Although they are still widely used, severe side effects and the emergence of drug-resistant trypanosomes have been reported, which hampers their efficacy. In particular, the emergence of multidrug-resistant trypanosome strains or animal trypanosomosis [15] has been reported in several locations in developing countries, including Mali [16], Mozambique [17], Cameroon [18], Burkina Faso [19,20], Ethiopia [21], Somalia [22], and Zambia [23]. Moreover, only a few drugs have been used for a long time for the treatment of human African trypanosomiasis (HAT). Because drugs against AAT and HAT are only administered by intramuscular and/or intravenous injections, medical or veterinary infrastructure is required to monitor their side effects among injected patients and infected animals, and to prevent infection with contaminated pathogens via reuse of syringes. Currently, fexinidazole is approved as the first oral drug for the treatment of HAT caused by *Trypanosoma brucei gambiense* [24,25]. Orally administered drugs have the advantage of easy intake without syringe use and reduce the risk of contamination. Therefore, the development of new orally administered drugs against animal trypanosomosis may have advantages in some husbandry systems.

Nitrofurantoin (NF), which is used as an antimicrobial agent for the treatment of human bacterial urinary tract infections, has been listed in the World Health Organization’s model list of essential medicines [26]. Nifurtimox, another nitrofuran derivative, was developed in the 1960s and has been used for the treatment of Chagas disease caused by *Trypanosoma cruzi*. Most recently, nifurtimox was evaluated in combination with eflornithine (NECT) for the treatment of late-stage HAT caused by *T. b. gambiense* [27,28,29].

Although NF has proven its anti-urinary tract infection effectiveness in pets, specifically in dogs [30] and cats [31], nitrofuran antibiotics have collectively been banned as livestock antibacterial treatments in the European Union countries since 1995 and by the US Food and Drug Administration (FDA) in 2002 due to carcinogenicity risks associated with the consumption of edible animal products, such as meat and milk [32,33].

We recently reported the potential for trypanocidal drug development of NF and its analogs through the evaluation of trypanocidal activity against trypanosomes in vitro [34]. Based on the preliminary results reported in our previous publication, the aim of the current study was to evaluate the in vivo dose-dependent treatment efficacy of orally administrated NF against animal trypanosomosis caused by *T. congolense*. The outcome would allow the establishment of an initial benchmark for in vivo trypanocidal efficacy for the comparison of future NF-related compounds. We herein report our findings.

## 2. Results

### Treatment Efficacy of NF against T. congolense-Infected Mice

**Figure 1:**
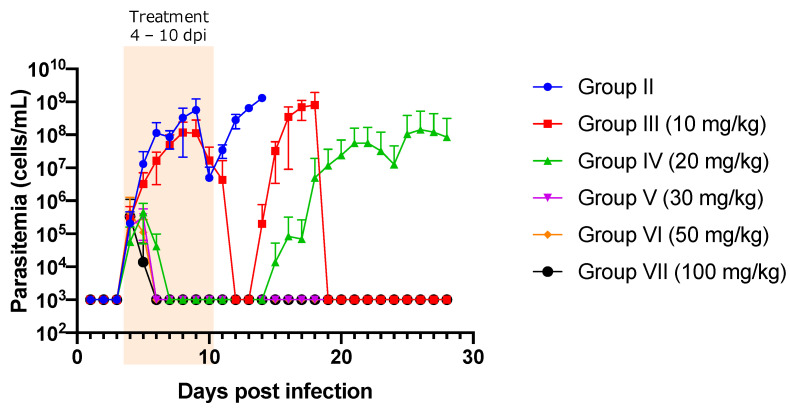
The evaluation of parasitemia in mice infected with *Trypanosoma congolense* and orally treated with different concentrations of nitrofurantoin. The Y-axis shows log 10 scale, and 10^3^ indicates no parasites in 100-fold diluted blood, as determined using a cell counting chamber. Significant suppression of parasitemia (*p* < 0.05) was observed on 6, 8, and 9 dpi in group III (10 mg/kg), and on 6 to 9 dpi in other groups in comparison with that in the control group (Group II) from days 6 to 9. The data are shown as the mean ± standard deviation values. Orange highlight: NF treatment from 4 dpi to 10 dpi.

A summary of the experiments is presented in Table 1. The prepatent period was days 3–4. Group I (non-treatment group) showed high levels of parasitemia and was euthanized within 14 days post infection (dpi). Parasitemia was significantly suppressed in group III (10 mg/kg) at 6, 8, 9, and 12–14 dpi compared to that in group II (non-treated group) (*p* < 0.05) (Figure 1). Similarly, significant suppression of parasitemia was observed from 6 to 9 dpi and from 12 to 14 dpi when NF was administered at doses higher than 20 mg/kg compared to that in the control group (*p* < 0.05) (Figure 1). Trypanosomes in 87.5% of the mice in group III (10 mg/kg) (7/8) and 100% of the mice in group IV (20 mg/kg) (8/8) completely disappeared from the peripheral blood, as confirmed by microscopic observation. However, relapsing parasitemia was noted at 14 dpi (1/7) and 15 dpi (6/7) in group III (10 mg/kg), and 15 dpi (1/2) and 19 dpi (1/2) in group IV (20 mg/kg) (Figure 1). In contrast, 12.5% of group III (10 mg/kg; 1/8), 75% of group IV (20 mg/kg; 6/8), and 100% of the mice treated with NF at >30 mg/kg (Group V, VI and VII) did not show parasitemia once trypanosomes had disappeared by treatment from the blood circulation (Figure 1). However, although trypanosomes could not be detected upon microscopic observation, they could be detected upon PCR in a surviving mouse at 28 dpi in group III (10 mg/kg) (Figure 1 and Supplementary Figure S1). In contrast, trypanosomes were detected by both microscopic observation and PCR in one surviving mouse at 28 dpi in group IV (20 mg/kg; 1/7, 14.29%) (Figure 1 and Supplementary Figure S1), while trypanosome infection was detected neither upon microscopic observation nor by PCR in the other mice in group IV (20 mg/kg; 6/7, 85.71%) and mice in the group treated with >30 mg/kg at 28 dpi (Figure 1 and Supplementary Figure S1). Sub-microscopic infection levels of trypanosomes in the animals from group III (10 mg/kg; 1/1, 100%) and group IV (20 mg/kg; 1/7, 14.29%) were confirmed in the mice injected with the blood collected from these animals at 28 dpi. On the other hand, no trypanosome infection was observed in the mice injected with blood collected from the groups treated with NF at doses higher than 30 mg/kg at 28 dpi (Table 1). 

**Figure 2:**
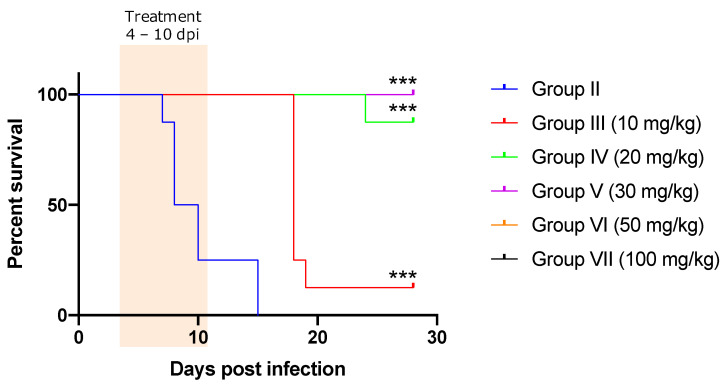
Survival curves of the mice infected with *Trypanosoma congolense* and orally treated with different concentrations of nitrofurantoin. The survival rate was significantly different from that of the control group (Group II). ***: *p* < 0.001 (log-rank test). Orange highlight: NF treatment from 4 dpi to 10 dpi.

**Figure 3:**
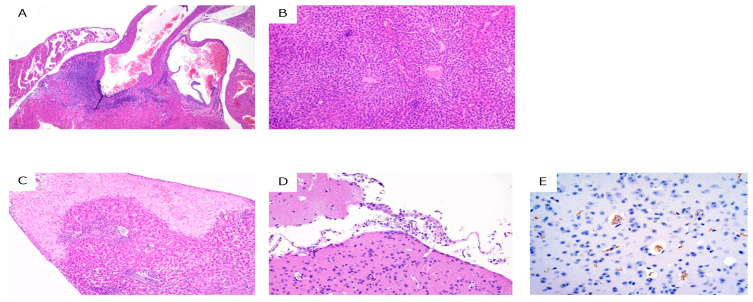
Histopathological analysis of trypanosome-infected mice at 28 dpi. (**A**) necrotizing arteritis in the heart (hematoxylin and eosin (HE) staining) in group III; (**B**) vasculitis and perivasculitis in the liver (HE staining) in group III; (**C**) focal necrosis in the liver (HE staining) in group IV; (**D**) meningoencephalitis in the brain (HE staining) in group IV; and (**E**). trypanosomes in the brain (immunohistochemical staining for trypanosomes, brown) in group IV.

Survival was significantly prolonged in all the treated groups (*p* < 0.001) compared to that in the control group (Figure 2). Body weights were not significantly different between the control and treatment groups, except at 3 dpi in group VII (100 mg/kg; *p* = 0.02), 8 dpi in group II (control group; *p* = 0.04), and 17 and 18 dpi in group III (10 mg/kg; *p* = 0.03 and *p* = 0.008, respectively). Several hematological parameters showed significant differences at several dpi in various groups (Supplementary Table S1). A summary of the histopathological analysis is presented in Supplementary Table S2. Necropsy was performed on only one surviving mouse in group III (10 mg/kg) at 28 dpi. Although the parasitemia in the mouse was sub-microscopic, severe necrotic vasculitis in the aortic root, mild vasculitis/perivasculitis in the liver, and lymphocytic panniculitis were observed. In addition, PCR was positive using DNA extracted from several tissues of the mouse as a template (Supplementary Table S2). In group IV (20 mg/kg), seven out of eight mice survived and only one mouse showed severe parasitemia in the peripheral blood at 28 dpi upon microscopic observation. Gross examination revealed splenomegaly in the mice with parasitemia. Histopathological analysis revealed focal to coalescing necrosis and severe vasculitis in the spleen and liver. Moreover, mild non-suppurative meningoencephalitis, myocarditis, and interstitial pneumonia were observed. IHC revealed that *T. congolense* trypanosomes were localized in the blood vessels, subarachnoid space, ventricles, and brain parenchyma in the mice. In groups V (30 mg/kg), VII (50 mg/kg), and VIII (100 mg/kg), all mice survived and did not show parasitemia. The histopathological examination showed mild pathological changes, such as vasculitis, perivasculitis, and pericholangitis, in the liver only (Figure 3 and Supplementary Table S2). The PCR results were negative, except for one mouse in group V (30 mg/kg).

## 3. Discussion

In this study, we evaluated the efficacy of orally administered NF against *T. congolense* infection in vivo by using a mouse model. We found that orally administered NF at doses higher than 30 mg/kg was completely efficacious based on the fact that parasitemia was observed neither by microscopic observation nor by PCR in the peripheral blood of the treated mice. Necropsy of these animals showed only mild histopathological changes in the organs, and the naïve mice injected with the blood from infected mice did not show parasitemia, confirming reliable treatment of the disease and complete clearance of the parasite. Severe pathological changes were observed in the trypanosome-positive mice upon PCR-based confirmation. In addition, IHC revealed many trypanosomes in the mice (group IV, 20 mg/kg), which showed the most severe pathological changes and parasitemia at 28 dpi. Moreover, although trypanosomes were not detected in tissue samples upon PCR, mild pathological changes, such as vasculitis, pericholangitis, perivasculitis, and focal necrosis, were observed in the liver tissue of several mice in the groups treated with doses higher than 30 mg/kg at 28 dpi. These pathological changes might be due to the high levels of parasitemia observed in the early phase at 4–6 dpi and were related to tissue recovery. These results clearly showed that the degree of pathological severity was related to the degree of severity of trypanosome infection. 

The NF concentration in the plasma per oral administration in a rodent model might be higher than the IC_50_ against *T. congolense*. Mario et al. [35] showed that the NF concentration in plasma was >10 µg/mL after 30 min of oral administration of 20 mg/kg NF in a mouse model. In addition, Wang and Morris [36] revealed that NF concentration in plasma had reached the mean highest point (C_max_) of 1.01 ± 0.47 µg/mL and the half-life (T_1/2_) was 166 ± 67.4 min per oral administration of 10 mg/kg NF in a rat model. These results indicated that the 30 mg/kg per oral administration in the current study might induce a sufficiently high concentration to kill *T. congolense* in the bloodstream. The pharmacokinetic parameters of orally administered NF (50–300 mg/head) in humans were as follows: C_max_, 0.21–3.7 µg/mL; T_max_, 0.5–5.0 h; and t_1/2_, 0.9–6.3 h [37,38]. Our previous in vitro study showed that the IC_50_s against other trypanosomes (except *T. congolense*) were higher than 300 ng/mL (0.3 µg/mL) [34]. Therefore, the combination of NF with other trypanocidal drugs such as eflornithine might provide a good treatment effect against other trypanosome infections as well.

NF is a promising drug against trypanosome infections because it is widely used for the treatment of urinary tract infections, and it is included in the WHO model list of essential medicines. On the other hand, it was reported in Algeria and Zambia that NF-resistant bacteria were isolated from pastoral cattle [39,40]. However, NF resistance may not emerge frequently because the ratio of NF-resistant *Escherichia coli* isolates in European countries was low compared to that of other antibiotics [41,42]. To prevent the induction and spread of NF-resistant bacteria, veterinarians have to control the treatment dose when treating AAT and survey NF-resistant bacteria. In addition, the risk of occurrence of NF-resistant bacteria, carcinogenicity and genotoxicity are considered as risks attributed to residual NF metabolites in livestock products; therefore, the use of NF in domestic animals is banned in many countries (chromatographic detection of nitrofurans in foods of animal origin). Hence, the development of NF-related compounds with low toxicity, and more cost-effective than NF, are required for anti-AAT treatment in domestic animals [1]. It is noteworthy to indicate that the collective ban of nitrofuran antibiotics is not due to the toxicity induced by the 5-nitrofuran moiety but rather the hydrazone metabolite [43]. Indeed, the chemotherapeutic effects of these drugs are linked to the 5-nitrofuran moiety present in their structures.

In contrast, the toxic properties (carcinogenicity and mutagenicity) have been attributed to the side chain linked to the moiety and, hence, to their metabolites, e.g., nitrofurazone’s metabolite is the toxic semicarbazide. In other words, a new compound that has the 5-nitrofuran scaffold in its structure may possess the therapeutic benefit of nitrofurantoin but lack the intolerable toxicity. This hypothesis explains the recent surge of interest in nitroaromatic compounds as anti-infective. Therefore, we intend to use it in future investigations into new veterinary medicines related to nitrofurantoin. Furthermore, the biological properties of NF emanate from the production of reactive oxygen species and, ultimately, oxidative stress, owing to its 5-nitrofuran pharmacophore. There are several redox-active drugs currently in clinical trials, e.g., ferrocifen (anticancer), ferroquine (antimalarial), or other compounds such as quinones [44] and anthraquinones [45] that are related to nitrofurantoin in a shared mechanism but do not exhibit the toxicity of nitrofurantoin. Therefore, these compounds related to NF through the mechanism of oxidative stress may also be considered for investigation in the search for new veterinary medicines.

In conclusion, oral administration of NF (>30 mg/kg) may be used as an initial benchmark for the trypanocidal treatment of AAT caused by *T. congolense* for nitrofuran and redox-active derivatives. However, these are preliminary data in the treatment potential of NF for the development of new trypanocidal drugs based on NF-related compounds; therefore, there is a need for additional requirements, such as these compounds being cost-effective and possessing lower toxicity in vitro and in vivo. In addition, we have to reveal treatment efficacy against animal trypanosomoses in a large animal model before establishing a definitive conclusion on the suitability of these compounds to act as new trypanocidal agents. The mode of action of more cost-effective and safer NF-analogs against *T. congolense*, and the pharmacodynamics of orally administered analogs in domestic animals, which are the main targets of AAT, should be analyzed for the treatment of AAT in these animals in endemic countries in the future.

## 4. Materials and Methods

### 4.1. Animal Experiments

Healthy female 8-week-old BALB/c mice (CLEA Japan Inc., Tokyo, Japan) were used in this study. All animals had ad libitum access to normal chow and water. The experiment was approved by the Animal Ethics Committee of the Obihiro University of Agriculture and Veterinary Medicine (Approval No. 20-9). The virulent *T. congolense* IL3000 strain was propagated in a mouse and used for infection. The parasites were passaged once in a mouse prior to the experiment. The experimental mice were intraperitoneally infected with 100 μL of *T. congolense* at 1 × 10^3^ cells/mouse inoculated with 100 μL of phosphate-buffered saline with 10% glucose (PSG). The mice were randomly divided into the following seven groups of four mice each: group I (no infection group), the mice were not infected and treated with only solvent (10% DMSO-corn oil); group II (control group), the mice were infected and treated with only solvent; groups III, IV, V, VI, and VII (the test groups), the mice were infected and orally treated using a feeding needle with 10, 20, 30, 50, and 100 mg/kg NF, respectively. Treatment was initiated at 4 dpi after the confirmation of trypanosome infection in the bloodstream by using the wet smear technique and was continued for seven consecutive days. The treatments were freshly prepared daily. The surviving mice were euthanized under anesthesia, and organs were collected for pathological and molecular parasitological analyses at 28 dpi. The experiments were conducted in duplicate (eight mice in groups II–VII and 12 mice in group I). To evaluate the infectivity of blood samples with sub-microscopic and sub-PCR detection levels of trypanosome infection, blood was collected from the surviving mice at 28 dpi and injected into naïve mice followed by parasitemia observation.

### 4.2. Evaluation of Parasitemia

To detect trypanosome infection, the wet blood smear technique was used. When the mice showed high levels of parasitemia, blood was diluted at an appropriate concentration using PSG and the number of trypanosomes in blood was determined using a Neubauer Improved Cell Counting Chamber (FUKAE-KASEI CO., LTD, Hyogo, Japan) to evaluate parasitemia. In addition to these parasitological methods, trypanosome infection was evaluated using PCR. Briefly, mouse blood samples collected from the tail vein were diluted 20 times with distilled water, and the diluted blood was directly used in PCR as a template. In addition, total DNA was extracted from tissue samples collected from euthanized mice by using a Qiagen DNA mini kit (Qiagen, Hilden, Germany) in accordance with the manufacturer’s instructions. TCS-PCR was used to detect *T. congolense* by using the TCS primers [46]. Briefly, a reaction mixture (10 μL) containing 1 µL of the sample, 5 µL of 2× MightyAmp buffer Ver. 3 (Takara Bio Inc., Shiga, Japan), 0.3 µM of each forward and reverse primer, 1 µL of 10× Additive for High Specificity (Takara Bio Inc.), 0.2 µL of MightyAmp DNA polymerase Ver.3 (Takara Bio Inc.), and 2.2 μL of double distilled water were prepared for each PCR assay. The PCR cycling conditions were as follows: an initial pre-denaturation step at 98 °C for 2 min; 35 cycles of denaturation at 98 °C for 10 s, annealing at 66 °C for 15 s, and extension at 68 °C for 10 s, per the manufacturer’s protocol.

### 4.3. Hematological Parameters

Blood samples were collected from the tail vein of the mice and subjected to a blood count by using an automatic hematology analyzer (Celltac α, Nihon Kohden, Tokyo, Japan). The red blood cell count, hematocrit, mean corpuscular hemoglobin, hemoglobin, white blood cell count, mean corpuscular volume, mean corpuscular hemoglobin concentration, and platelet count were analyzed.

### 4.4. Histopathological Analyses

The liver, spleen, kidneys, heart, lung, brain, and adipose tissue were collected, fixed in 10% neutral buffered formalin, and subjected to histopathological analysis. Samples were processed using standard procedures, and then, two serial sections for all the examined mice were prepared from formalin-fixed paraffin-embedded tissue. The sections were stained with hematoxylin and eosin (HE) and for immunohistochemistry (IHC) with antibodies against α-tubulin of *T. congolense* [47]. For signal detection, the Envision system (Agilent, Santa Clara, CA, USA) with diaminobenzidine (DAB) as the substrate was used. IHC sections were counterstained with hematoxylin.

### 4.5. Statistical Analysis

The results are expressed as the mean ± standard deviation (SD) values of the number of repeated trials indicated in each experiment. One-way analysis of variance (ANOVA) and Dunnett’s test were used for comparisons between the no infection (group I) and the other groups (group II–VII) for body weight and blood parameters, or between the control (group II) and treated groups (group III–VII) for parasitemia in the acute phase. Survival curves were constructed using the Kaplan–Meier method, and the curves were compared using a log-rank test. All data were compiled using GraphPad Prism software (version 8.0, GraphPad Software Inc., San Diego, CA, USA). Statistical significance was set at *p* < 0.05.

## Figures and Tables

**Table 1:** Summary of the experiment.

Dose (mg/kg)	Total Mice Number	Survived Mice (%)	Euthanized Mice (%)	Mean Day of Survival in Euthanized Mice ± SD	Relapsed Mice (%)	Mean Day of Relapse ± SD	Re-infected Mice Numbers	Infected Mice in Re-Infection	% Infected in Re-Infected Mice
Group II (Non treated)	8	0 (0.00)	8 (100)	9.15 ± 3.18	0 (0.00)	14.85 ± 0.37	0	NA	NA
Group III (10 mg/kg)	8	1 (12.50)	7 (87.50)	17.14 ± 0.38	7 (87.5)	14.85 ± 0.38	1	1	100
Group IV (20 mg/kg)	8	7 (87.50)	1 (12.50)	23.00	2 (25.00)	17.00 ± 2.83	7	1	14.29
Group V (30 mg/kg)	8	8 (100)	0 (0.00)	ND	0 (0.00)	ND	4 *	0	0.00
Group VI (50 mg/kg)	8	8 (100)	0 (0.00)	ND	0 (0.00)	ND	4 *	0	0.00
Group VII (100 mg/kg)	8	8 (100)	0 (0.00)	ND	0 (0.00)	ND	4 *	0	0.00

Summary of treatment efficacy of nitrofurantoin against animal African trypanosomosis caused by *Trypanosoma congolense* in mice following the drug’s oral administration at different doses. NA: Not analyzed. ND: Not determined. *: Re-infection by the injection of the blood collected from two surviving mice into a naïve mouse.

## Data Availability

Not applicable.

## Supplementary Materials

The following supporting information can be downloaded at: https://www.mdpi.com/article/10.3390/pathogens11030331/s1, Figure S1: Trypanosome infection were evaluated by blood direct PCR in the experiment period. PCR detection (%) was calculated as positive case divided by tested mice in each group; Table S1: Summary of blood parameters; Table S2: Summary of histopathological analysis.
