# Supplementary material for: Therapeutic Efficacy of Orally Administered Nitrofurantoin against Animal African Trypanosomosis Caused by Trypanosoma congolense Infection
Source: Pathogens. 2022 Mar 9;11(3):331. doi: 10.3390/pathogens11030331 (PMC8956101; doi:10.3390/pathogens11030331)
Supplement: Supplementary file 1 [file pathogens-11-00331-s001.zip › pathogens-1555758-supplementary.pdf]

Supplement Table S1: Summary of blood parameters

**Analyzed mice number**	**Group I**	**Group II (Non treated)**	**Group III (10 mg/kg)**	**Group IV (20 mg/kg)**	**Group V (30 mg/kg)**	**Group VI (50 mg/kg)**	**Group VII (100 mg/kg)**
Pre	12	8	8	8	8	8	8
Day2	12	8	8	8	8	8	8
Day4	12	8	8	8	8	8	8
Day7	12	6	8	8	8	8	8
Day9	12	2	8	8	8	8	8
Day11	12	2	8	8	8	8	8
Day14	12		8	8	8	8	8
Day16	12		8	8	8	8	8
Day18	12		1	8	8	8	8
Day25	12		1	7	8	8	8

WBC ($10^2/\mu L$)	Group I	Group II (Non treated)	Group III (10 mg/kg)	Group IV (20 mg/kg)	Group V (30 mg/kg)	Group VI (50 mg/kg)	Group VII (100 mg/kg)
Pre	160.17 ± 42.00	155.00 ± 56.15	133.00 ± 22.53	187.13 ± 32.64	138.00 ± 18.36	122.75 ± 35.61	137.25 ± 57.10
Day2	156.50 ± 35.57	114.38 ± 44.14	80.63 ± 24.53***	108.25 ± 19.16*	96.38 ± 30.39**	118.63 ± 34.72	104.5 ± 16.64*
Day4	159.58 ± 34.14	133.63 ± 19.26	121.00 ± 22.74	146.63 ± 25.09	133.75 ± 20.48	137.88 ± 19.19	140.75 ± 43.46
Day7	146.83 ± 31.37	111.00 ± 20.14	134.63 ± 37.66	155.50 ± 38.41	111.75 ± 16.08	165.50 ± 24.11	184.13 ± 52.40
Day9	142.42 ± 34.61	288.50 ± 31.82****	206.38 ± 64.80**	100.13 ± 20.02	71.13 ± 10.62***	139.75 ± 20.39	186.13 ± 41.59
Day11	120.58 ± 32.80	169.50 ± 58.69	203.63 ± 131.5****	103.38 ± 37.43	78.75 ± 13.33	169.88 ± 31.93*	132.25 ± 23.80
Day14	169.42 ± 22.83		125.25 ± 50.23*	126.63 ± 28.27*	127.63 ± 37.42*	149.00 ± 52.04	135.75 ± 55.92
Day16	122.42 ± 39.33		107.63 ± 29.83	64.00 ± 8.05**	66.63 ± 16.28**	149.75 ± 16.52	128.25 ± 15.42
Day18	170.42 ± 39.65		133	139.50 ± 42.00	142.13 ± 20.10	165.50 ± 23.15	134.50 ± 22.47
Day25	144.00 ± 23.09		148	224.29 ± 74.37***	158.25 ± 40.35	162.00 ± 23.32	144.13 ± 22.77

RBC ($10^4/\mu L$)	Group I	Group II (Non treated)	Group III (10 mg/kg)	Group IV (20 mg/kg)	Group V (30 mg/kg)	Group VI (50 mg/kg)	Group VII (100 mg/kg)
Pre	869.33 ± 68.45	884.88 ± 84.08	883.13 ± 61.80	917.63 ± 76.44	946.88 ± 32.99	915.75 ± 88.55	922.63 ± 50.71
Day2	870.92 ± 65.62	923.13 ± 51.58	797.13 ± 162.68	962.75 ± 34.12	986.88 ± 54.31*	868.25 ± 85.18	929.00 ± 107.45
Day4	812.26 ± 265.90	954.38 ± 153.17**	927.75 ± 78.91*	928.25 ± 90.12*	941.13 ± 40.36*	878.50 ± 68.21	915.63 ± 77.19
Day7	925.58 ± 34.28	853.33 ± 60.40	842 ± 78.55	872.50 ± 81.12	930.63 ± 34.00	861.88 ± 64.93	899.13 ± 112.14
Day9	927.00 ± 77.09	771.50 ± 34.65	747.88 ± 83.87***	945.63 ± 80.62	966.25 ± 48.00	850.88 ± 93.20	881.88 ± 119.53
Day11	882.50 ± 70.16	838.00 ± 121.62	837.63 ± 84.56	880.38 ± 85.38	977.38 ± 50.13	909.88 ± 46.96	911.63 ± 60.85
Day14	902.00 ± 93.92		909.25 ± 122.38	851.38 ± 39.49	878.88 ± 72.76	944.00 ± 54.30	951.50 ± 41.65
Day16	857.33 ± 60.51		819.88 ± 108.51	926.00 ± 69.92	896.63 ± 84.69	924.125 ± 62.11	883.13 ± 85.71
Day18	902.75 ± 42.81		865	902.38 ± 80.61	857.25 ± 59.16	939.13 ± 61.20	910.38 ± 35.24

Day25	904.50 ± 64.17		816	874.14 ± 77.38	919.88 ± 97.21	936.75 ± 64.06	913.13 ± 74.17
HGB (g/dL)	Group I	Group II	Group III (10 mg/kg)	Group IV (20 mg/kg)	Group V (30 mg/kg)	Group VI (50 mg/kg)	Group VII (100 mg/kg)
Pre	12.87 ± 0.83	12.99 ± 1.33	12.96 ± 0.71	13.35 ± 1.10	13.71 ± 0.49	13.41 ± 1.31	13.50 ± 0.67
Day2	12.90 ± 0.97	13.51 ± 0.68	11.81 ± 2.35	14.10 ± 0.73	14.53 ± 0.80*	12.96 ± 1.23	13.86 ± 1.55
Day4	12.87 ± 1.31	13.94 ± 2.69	13.31 ± 1.17	13.51 ± 1.33	13.41 ± 0.63	12.71 ± 0.77	13.39 ± 1.21
Day7	13.51 ± 0.63	12.35 ± 0.87	12.33 ± 0.99	12.46 ± 1.22	13.35 ± 0.46	12.51 ± 1.03	13.01 ± 1.61
Day9	13.69 ± 1.21	11.15 ± 0.35*	10.64 ± 1.25****	13.80 ± 1.26	13.93 ± 0.64	12.04 ± 1.15*	12.86 ± 1.68
Day11	12.87 ± 1.01	11.90 ± 1.84	11.89 ± 1.08	12.43 ± 1.18	13.96 ± 0.72	12.96 ± 0.68	13.19 ± 1.05
Day14	13.23 ± 0.88		12.73 ± 1.59	11.94 ± 0.52*	12.44 ± 1.10	13.31 ± 0.70	13.56 ± 0.56
Day16	12.56 ± 0.87		11.54 ± 1.35	13.06 ± 0.78	12.98 ± 1.23	13.01 ± 0.95	12.49 ± 1.31
Day18	13.15 ± 0.69		12.2	12.65 ± 1.04	12.20 ± 1.01	13.19 ± 0.85	12.91 ± 0.51
Day25	13.06 ± 0.91		10.8	12.10 ± 1.17	12.96 ± 1.46	13.03 ± 0.89	12.83 ± 1.00
HCT (%)	Group I	Group II (Non treated)	Group III (10 mg/kg)	Group IV (20 mg/kg)	Group V (30 mg/kg)	Group VI (50 mg/kg)	Group VII (100 mg/kg)
Pre	40.23 ± 3.10	40.76 ± 4.14	41.04 ± 2.72	42.68 ± 3.51	43.94 ± 1.29	42.61 ± 4.22	42.81 ± 2.48
Day2	40.08 ± 2.74	42.25 ± 2.72	37.09 ± 7.34	44.51 ± 2.23*	45.68 ± 2.59**	40.23 ± 4.07	43.10 ± 4.96
Day4	40.26 ± 4.47	43.19 ± 6.71	42.44 ± 3.62	42.33 ± 4.13	42.61 ± 1.70	40.04 ± 3.12	41.96 ± 3.54
Day7	42.73 ± 1.61	38.32 ± 2.54	38.63 ± 3.35	39.85 ± 3.73	42.26 ± 1.40	39.45 ± 3.11	41.15 ± 5.11
Day9	42.79 ± 3.22	35.35 ± 0.92*	34.71 ± 4.51****	42.95 ±3.60	44.04 ± 2.10	38.79 ± 4.05	40.51 ± 5.36
Day11	40.36 ± 2.87	37.75 ± 6.34	39.63 ± 3.28	39.79 ± 3.47	43.99 ± 2.19	41.25 ± 2.22	41.56 ± 2.82
Day14	41.45 ± 2.74		42.03 ± 5.00	38.49 ± 1.60	39.74 ± 3.39	42.95 ± 2.35	43.55 ± 1.94
Day16	39.37 ± 2.55		37.04 ± 4.39	41.85 ± 2.66	41.04 ± 4.12	41.75 ± 2.87	40.30 ± 3.87
Day18	41.43 ± 1.83		42	40.95 ± 3.57	39.04 ± 2.73	42.48 ± 2.86	41.41 ± 1.70
Day25	41.45 ± 2.81		38.3	38.99 ± 3.64	41.58 ± 4.35	41.86 ± 2.73	41.20 ± 3.23
MCV (fL)	Group I	Group II (Non treated)	Group III (10 mg/kg)	Group IV (20 mg/kg)	Group V (30 mg/kg)	Group VI (50 mg/kg)	Group VII (100 mg/kg)
Pre	46.27 ± 0.45	46.05 ± 0.52	46.49 ± 1.05	46.50 ± 0.83	46.41 ± 0.51	46.54 ± 0.47	46.38 ± 0.46
Day2	46.05 ± 0.69	45.76 ± 0.53	46.58 ± 0.91	46.21 ± 0.81	46.28 ± 0.36	46.31 ± 0.60	46.41 ± 0.30
Day4	46.28 ± 0.38	45.28 ± 0.51**	45.74 ± 1.13	45.59 ± 0.93	45.28 ± 0.36**	45.56 ± 0.49	45.83 ± 0.42
Day7	46.17 ± 0.68	44.92 ± 0.60**	45.89 ± 1.04	45.68 ± 0.84	45.43 ± 0.47	45.78 ± 0.40	45.76 ± 0.29
Day9	46.19 ± 0.89	45.85 ± 0.92	46.34 ± 1.16	45.45 ± 0.92	45.56 ± 0.47	45.63 ± 0.41	45.95 ± 0.42
Day11	45.78 ± 0.70	44.95 ± 1.20	47.40 ± 1.07****	45.26 ± 0.93	45.01 ± 0.33	45.34 ± 0.55	45.60 ± 0.29
Day14	45.98 ± 0.70		46.39 ± 2.04	45.23 ± 0.89	45.20 ± 0.50	45.50 ± 0.53	45.78 ± 0.37
Day16	45.95 ± 0.49		45.28 ± 2.08	45.23 ± 1.04	45.74 ± 0.51	45.18 ± 0.47	45.64 ± 0.39
Day18	45.89 ± 0.49		48.6*	45.38 ± 0.97	45.53 ± 0.38	45.23 ± 0.56	45.49 ± 0.28
Day25	45.83 ± 0.52		46.9	44.59 ± 1.02*	45.20 ± 0.74	44.69 ± 0.54*	45.14 ± 0.45

MCH (pg)	Group I	Group II (Non treated)	Group III (10 mg/kg)	Group IV (20 mg/kg)	Group V (30 mg/kg)	Group VI (50 mg/kg)	Group VII (100 mg/kg)
Pre	14.82 ± 0.32	14.66 ± 0.33	14.70 ± 0.43	14.56 ± 0.27	14.51 ± 0.27	14.65 ± 0.30	14.65 ± 0.23
Day2	14.81 ± 0.45	14.65 ± 0.35	14.85 ± 0.40	14.65 ± 0.33	14.70 ± 0.19	14.91 ± 0.27	14.91 ± 0.25
Day4	14.80 ± 0.43	14.51 ± 0.53	14.35 ± 0.64*	14.55 ± 0.24	14.25 ± 0.23**	14.50 ± 0.45	14.64 ± 0.41
Day7	14.61 ± 0.35	14.48 ± 0.16	14.65 ± 0.27	14.25 ± 0.24	14.36 ± 0.18	14.53 ± 0.39	14.46 ± 0.22
Day9	14.77 ± 0.32	14.45 ± 0.21	14.23 ± 0.45**	14.61 ± 0.38	14.44 ± 0.21	14.16 ± 0.38**	14.59 ± 0.41
Day11	14.58 ± 0.26	14.20 ± 0.14	14.23 ± 0.39	14.13 ± 0.23*	14.29 ± 0.60	14.24 ± 0.24	14.46 ± 0.23
Day14	14.67 ± 0.35		14.03 ± 0.48****	14.05 ± 0.32****	14.14 ± 0.15***	14.13 ± 0.36***	14.26 ± 0.21*
Day16	14.65 ± 0.30		14.10 ± 0.47***	14.11 ± 0.30***	14.46 ± 0.11	14.08 ± 0.25***	14.13 ± 0.21***
Day18	14.57 ± 0.32		14.1	14.03 ± 0.25***	14.21 ± 0.27*	14.04 ± 0.23***	14.18 ± 0.19*
Day25	14.44 ± 0.19		13.2***	13.84 ± 0.29***	14.09 ± 0.34*	13.91 ± 0.28***	14.05 ± 0.19*

MCHC (pg)	Group I	Group II (Non treated)	Group III (10 mg/kg)	Group IV (20 mg/kg)	Group V (30 mg/kg)	Group VI (50 mg/kg)	Group VII (100 mg/kg)
Pre	32.03 ± 0.67	31.86 ± 0.57	31.63 ± 0.95	31.30 ± 0.43	31.19 ± 0.52	31.49 ± 0.40	31.58 ± 0.71
Day2	32.19 ± 0.83	32.01 ± 0.78	31.84 ± 0.75	31.66 ± 0.27	31.83 ± 0.28	32.25 ± 0.51	32.19 ± 0.59
Day4	32.01 ± 0.88	32.11 ± 1.27	31.38 ± 1.27	31.91 ± 0.27	31.48 ± 0.44	31.79 ± 0.96	31.90 ± 1.05
Day7	31.61 ± 0.56	32.23 ± 0.39	31.91 ± 0.49	31.26 ± 0.41	31.59 ± 0.38	31.71 ± 0.67	31.61 ± 0.31
Day9	31.98 ± 0.69	31.55 ± 0.21	30.68 ± 0.78***	32.14 ± 0.65	31.63 ± 0.27	31.06 ± 0.65*	31.75 ± 0.70
Day11	31.88 ± 0.58	31.55 ± 0.49	29.99 ± 0.77****	31.21 ± 0.39	31.78 ± 1.26	31.44 ± 0.46	31.70 ± 0.52
Day14	31.92 ± 0.69		30.26 ± 1.20****	31.01 ± 0.22**	31.31 ± 0.57	31.00 ± 0.51**	31.15 ± 0.40*
Day16	31.90 ± 0.58		31.19 ± 0.90*	31.24 ± 0.31	31.63 ± 0.36	31.16 ± 0.40*	30.96 ± 0.31**
Day18	31.73 ± 0.74		29.00****	30.90 ± 0.41**	31.23 ± 0.63	31.08 ± 0.22	31.19 ± 0.53
Day25	31.49 ± 0.58		28.20****	31.03 ± 0.40	31.16 ± 0.60	31.13 ± 0.47	31.13 ± 0.35

PLT (10^4/μL)	Group I	Group II (Non treated)	Group III (10 mg/kg)	Group IV (20 mg/kg)	Group V (30 mg/kg)	Group VI (50 mg/kg)	Group VII (100 mg/kg)
Pre	89.07 ± 11.68	86.14 ± 8.51	76.08 ± 12.60	87.00 ± 15.36	80.45 ± 8.21	84.69 ± 8.31	87.06 ± 11.58
Day2	91.13 ± 9.29	85.85 ± 8.34	68.56 ± 7.37***	78.28 ± 13.66	75.46 ± 8.29*	81.10 ± 8.03	75.49 ± 10.04*
Day4	88.93 ± 7.60	96.59 ± 20.58	80.21 ± 11.45	83.91 ± 9.80	87.41 ± 5.64	77.28 ± 16.35	81.23 ± 15.52
Day7	99.19 ± 10.13	92.38 ± 35.46	56.79 ± 19.09****	84.21 ± 10.48*	92.38 ± 11.91	87.63 ± 7.18	92.64 ± 5.62
Day9	97.83 ± 12.31	53.05 ± 13.22****	54.06 ± 15.59****	78.71 ± 6.21**	81.64 ± 9.73*	89.08 ± 11.51	95.10 ± 12.12
Day11	88.97 ± 11.00	48.85 ± 2.33***	71.64 ± 18.38*	88.48 ± 14.26	88.94 ± 7.99	93.74 ± 13.87	93.04 ± 8.77
Day14	90.93 ± 6.16		83.89 ± 17.10	89.40 ± 9.89	95.48 ± 15.07	83.83 ± 13.36	96.49 ± 12.58
Day16	84.89 ± 10.01		89.75 ± 22.04	82.53 ± 10.59	87.19 ± 8.62	87.65 ± 11.62	83.18 ± 7.31
Day18	94.73 ± 7.27		63.3	78.58 ± 18.47*	87.79 ± 9.43	94.15 ± 17.73	92.54 ± 8.80
Day25	91.15 ± 8.32		68.9	91.47 ± 9.38	81.91 ± 10.45	82.11 ± 11.63	83.34 ± 12.63

WBC: white blood cell, RBC: red blood cell, HGB: hemoglobin, HCT: hematocrit, MCV: mean cell volume, MCH: mean cell hemoglobin, MCHC: mean cell hemoglobin concentration, PLT: platelet.

*: $p < 0.05$, **: $p < 0.01$, ***: $p < 0.001$, ****: $p < 0.0001$.Supplement

Table S2: Summary of histopathological analysis

	**Group III (10 mg/kg)**		**Group IV (20 mg/kg)**		**Group V (30 mg/kg)**		**Group VI (50 mg/kg)**		**Group VII (100 mg/kg)**	
Mice number	1		7		8		8		8	
Tissue	Pathological [n, %]	PCR positive (%)	Pathological [n, %]	PCR positive (%)	Pathological [n, %]	PCR positive (%)	Pathological [n, %]	PCR positive (%)	Pathological [n, %]	PCR positive (%)
Liver	vasculitis(mild) [1, 100%]	1 (100)	vasculitis (mild) [3, 43%], focal necrosis, vasculitis/perivasculitis [2, 28.57%]	2 (28.57)	vasculitis (mild) [4, 50.00 %], pericholangtis (mild) [2, 25.00 %]	0 (0)	pericholangitis (mild) [1, 12.50 %]	0 (0)	vasculitis (mild) [2, 25.00 %], focal necrosis, vasculitis/perivasculitis [1, 12.5%]	0 (0)
Spleen		1 (100)	focal necrosis, splenomegaly [1, 14.29 %]	2 (28.57)		0 (0)		0 (0)		0 (0)
Kidny		1 (100)	vasculitis/perivasculitis [1, 14.29 %]	3 (42.86)		0 (0)		0 (0)		0 (0)
Heart	necrotising artelitis in the aortic root [1, 100%]	1 (100)	myocarditis [1, 14.29 %]	4 (57.14)		1 (12.5)		0 (0)		0 (0)
Lung		1 (100)	interstitial neumonia (mild) [1, 14.29 %]	4 (57.14)		1 (12.5)		0 (0)		0 (0)
Fat	lymphocitic panniculitis [1, 100%]	1 (100)		4 (57.14)		0 (0)		0 (0)		0 (0)
Blain		1 (100)	non-suppurative meningoencephalitis [1, 14.29 %]	3 (42.86)		0 (0)		0 (0)		0 (0)

Trypanosome was detected only in a mouse in group IV (20 mg/kg), which is shown parasitemia on 28 dpi by immunohistochemistry.
