# Supplementary figures and images for: Therapeutic Efficacy of Orally Administered Nitrofurantoin against Animal African Trypanosomosis Caused by Trypanosoma congolense Infection
Source: Pathogens. 2022 Mar 9;11(3):331. doi: 10.3390/pathogens11030331 (PMC8956101; doi:10.3390/pathogens11030331)

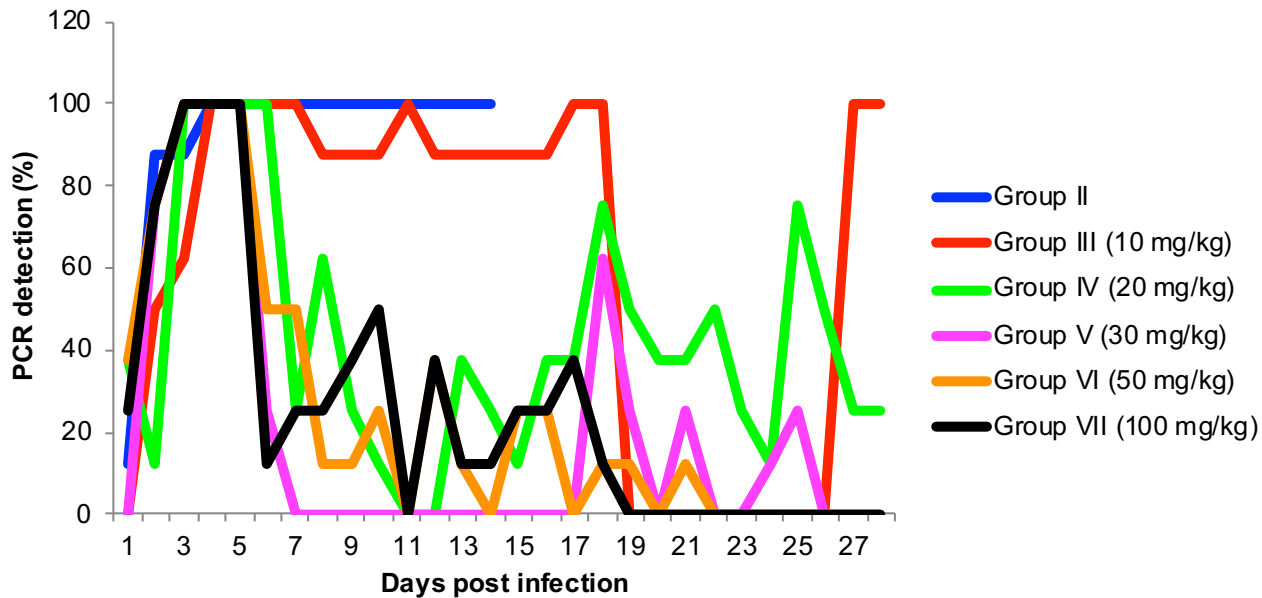

Supplement: Supplementary file 1 [file pathogens-11-00331-s001.zip › Figure S1.pdf]
